# Physiopathological Features in a Three-Dimensional In Vitro Model of Hepatocellular Carcinoma: Hypoxia-Driven Oxidative Stress and ECM Remodeling

**DOI:** 10.3390/cancers17183082

**Published:** 2025-09-21

**Authors:** Maria Giovanna Rizzo, Enza Fazio, Claudia De Pasquale, Emanuele Luigi Sciuto, Giorgia Cannatà, Cristiana Roberta Multisanti, Federica Impellitteri, Federica Gilda D’Agostino, Salvatore Pietro Paolo Guglielmino, Caterina Faggio, Sabrina Conoci

**Affiliations:** 1 Department of Chemical Sciences, Biological, Pharmaceutical and Environmental, University of Messina, Viale F. Stagno d’Alcontres, 31, 98166 Messina, Italy; emanueleluigi.sciuto@unime.it (E.L.S.); giorgia.cannata@studenti.unime.it (G.C.); sguglielm@unime.it (S.P.P.G.); sconoci@unime.it (S.C.); 2 Department of Mathematics and Computer Sciences, Physical Sciences and Earth Sciences, University of Messina, Viale F. Stagno d’Alcontres, 31, 98166 Messina, Italy; enza.fazio@unime.it; 3 Laboratory of Immunology, Department of Human Pathology DETEV, Messina University, 98166 Messina, Italy; claudia.depasquale@unime.it; 4 Department of Veterinary Sciences, University of Messina, Viale Giovanni Palatucci snc, 98168 Messina, Italy; cristiana.multisanti@studenti.unime.it (C.R.M.); federica.impellitteri@studenti.unime.it (F.I.); 5 Thoracic Surgery Unit, Papardo Hospital, 98158 Messina, Italy; feded789@gmail.com; 6 Department of Eco-Sustainable Marine Biotechnology, Stazione Zoologica Anton Dohrn, 80122 Naples, Italy; 7 Department of Chemistry “Giacomo Ciamician”, Bologna Univiversity, 40126 Bologna, Italy

**Keywords:** hepatocellular carcinoma (HCC), three-dimensional (3D) model, hypoxia, ECM remodeling, tumor microenvironment, pathophysiology

## Abstract

*Background*: Hypoxia is a hallmark of solid tumors, including hepatocellular carcinoma (HCC), where it drives oxidative stress and extracellular matrix (ECM) remodeling, promoting tumor invasion and metastasis. Investigating these mechanisms in patients remains challenging due to the complexity of the tumor microenvironment. *Methods*: We developed a scaffold-free three-dimensional (3D) spheroid model of HCC using human hepatocellular carcinoma HepG2 cells (ATCC HB-8065). To characterize hypoxia-driven processes, a multiparametric approach combining MTT assays for metabolic activity, confocal microscopy for viability and ECM organization, flow cytometry for apoptosis and ROS detection, qRT-PCR for gene expression, and FTIR spectroscopy for biochemical profiling were performed. *Results*: The 3D model exhibited progressive ROS accumulation, stabilization of HIF-1α, and metabolic reprogramming toward aerobic glycolysis. In parallel, ECM remodeling was evident, with increased expression of SPARC and FN1 and collagen fiber alignment, reflecting an invasive tumor phenotype. *Conclusions*: This scaffold-free 3D HCC model recapitulates key physiopathological features of tumor progression, providing a robust and physiologically relevant platform to investigate the hypoxia–ROS–ECM relationship and to support preclinical evaluation of targeted therapeutic strategies.

## Simple Summary

Hepatocellular carcinoma (HCC) ranks among the most common causes of cancer mortality worldwide. Traditional two-dimensional cultures fail to mimic the tumor environment. In this work, we developed a scaffold-free three-dimensional (3D) spheroid model of HCC using HepG2 cells (ATCC HB-8065) to investigate tumor physiology. Through metabolic, imaging, molecular, and spectroscopic analyses, we observed hypoxia-induced accumulation of reactive oxygen species (ROS), stabilization of HIF-1α, metabolic reprogramming toward glycolysis, and progressive ECM remodeling with increased SPARC and FN1 expression. This 3D model captures essential features of HCC progression and provides a reliable tool for studying tumor dynamics and testing new therapeutic strategies.

## 1. Introduction

Hepatocellular carcinoma (HCC) is the primary liver neoplasm and the leading cause of cancer death in some areas of the world. In 2018, HCC was considered a major health problem in Asia, with a case incidence of 72%, and Africa, with a lower prevalence in Europe and America [1]. The incidence of HCC is steadily increasing in many areas of the world, with an estimated over 900,000 new cases diagnosed annually, making it the sixth most common malignancy and the third leading cause of cancer death worldwide [2]. The main predisposing conditions include cirrhosis of the liver, which often results from chronic infections with hepatitis B (HBV) and C (HCV) viruses, alcohol abuse, metabolic dysfunction-associated steatotic liver disease (MASLD) [3], and exposure to toxic substances such as aflatoxin B1 [4]. Clinically, HCC is often asymptomatic in the early stages and is therefore usually diagnosed at an advanced stage, making it particularly insidious [5]. Despite advances in prevention techniques, screening, and new technologies for diagnosis and treatment, including surgical resection, liver transplantation, ablative therapy, and systemic treatments through immunotherapies, clinical responses are still limited, particularly in inoperable or relapsing cases [6], while the incidence and mortality continue to increase. This has augmented the focus on its molecular pathogenesis, epidemiology, and treatment [7,8]. In this context, there is a need for physiologically relevant experimental models to better understand tumor biology, identify new diagnostic and prognostic biomarkers, and effectively test innovative therapeutic strategies.

Traditional in vitro models, such as immortalized cell lines (HepG2 and Hep3B), although useful in some respects, present significant limitations in terms of representativeness of tumor complexity. Two-dimensional (2D) cultures cannot faithfully replicate the cell–cell and cell–matrix interactions observed in vivo [9,10]. In the context of hepatocellular carcinoma (HCC), there is increasing interest in three-dimensional models, such as organoids and spheroids, and in vivo systems that better mimic tissue architecture, cellular heterogeneity, and tumor microenvironment interactions [11,12]. Furthermore, the 3D model of HCC appears to be more widely used for the assessment of drug-induced liver injury (DILI), a primary concern during drug development, while also improving the understanding of the biology of Plasmodium liver stages [13,14]. The current 3D HepG2 models for hepatocellular carcinoma (HCC) show limited repeatability with natural extracellular matrix (ECM)-derived scaffolds [15,16,17,18].

Therefore, the most advanced models need to account for the dynamic characteristics of the tumor microenvironment, including hypoxia, which is one of the most significant pathophysiological features in solid tumors, such as HCC [19,20]. Hypoxia, defined as reduced oxygen availability compared to physiological levels, is a frequent condition in the tumor context due to irregular vascularization and rapid cell proliferation, which exceeds the blood/oxygen supply capacity [21,22]. In solid tumors such as HCC, large hypoxic areas are observed that actively contribute to neoplastic progression, treatment resistance, and selection of more aggressive cell subpopulations [23]. The principal molecular mediator of the hypoxia response is hypoxia-inducible factor 1-alpha (HIF-1α), a transcription factor that, under normoxic conditions, is rapidly degraded. Under hypoxia, this factor stabilizes and translocates into the nucleus, where it activates genes involved in proliferation, angiogenesis (e.g., VEGF), immune evasion, and metabolic reprogramming toward aerobic glycolysis [21,22,24].

These adaptations enable tumor cells to survive in hostile conditions and to acquire invasive and metastatic phenotypic characteristics [25]. Moreover, hypoxia is closely interconnected with oxidative stress, another key element in tumor biology. Limited oxygen increases the production of reactive oxygen species (ROS) through mitochondrial dysfunction. ROS, in turn, can stabilize HIF-1α, forming a positive feedback loop that intensifies the hypoxic response in HCC [26,27,28,29]. In hepatocellular carcinoma (HCC), unlike normal differentiated cells, cancer cells predominantly rely on aerobic glycolysis, an inefficient way to produce adenosine 5′-triphosphate (ATP). Following Otto Warburg’s early findings, HCC cells consume significantly more glucose than healthy liver cells and preferentially convert glucose to lactate via glycolysis, even when oxygen is present, a phenomenon known as the “Warburg effect”. This involves upregulation of glucose transporters like GLUT1 and the enzyme LDHA, leading to lactate accumulation in the tumor microenvironment [30,31]. Lactate acts as a signal metabolite, modulating gene expression through inhibition of histone deacetylases (HDACs), affecting the tumor epigenome [32], and may contribute to the stabilization of HIF-1α, enhancing hypoxia-like responses in some tumor contexts. Finally, a further key mechanism in tumor progression is extracellular matrix (ECM) remodeling, which evolves from a simple support structure into an active regulator of cell migration and invasiveness. Proteins such as fibronectin (FN1), which is often overexpressed in liver tumors, contribute to the modulation of integrins, adhesion, and cell motility [33,34,35]. Furthermore, SPARC has been shown to have a pro-invasive function in different tumor contexts, including HCC. Its functional role varies depending on the tumor type considered and the tumor environment, and in HCC, it facilitates interaction with the matrix and tissue degradation. Furthermore, SPARC promoted migration and epithelial–mesenchymal transition in HCC cells [36]. Thus, hypoxia, ROS accumulation, and ECM remodeling represent interconnected physiopathological mechanisms that drive HCC progression. Recent studies have highlighted how three-dimensional liver models can offer complementary perspectives: 3D HepG2 spheroids combined with duplex sequencing showed specific mutational signatures within two weeks [37]. In parallel, albumin-based biomimetic scaffolds and bioprinted liver constructs have demonstrated long-term functionality in culture [38,39], and liver-on-chip microfluidic systems have produced lobule-specific zonation and perfusion [40].

This study aims to develop a scaffold-free 3D model of hepatocarcinoma using the human hepatocellular carcinoma cell line HepG2 (ATCC HB-8065) (3D HCC model). The main goal of this work is to investigate, for up to 4 weeks, hypoxia-induced remodeling of the extracellular matrix (ECM). In particular, this study focuses on hypoxia-driven mechanisms, such as HIF-1α signaling, metabolic reprogramming, oxidative stress, and ECM alterations, as key factors in HCC progression. This model offers a robust platform for studying tumor physiology under hypoxia and for advancing applications in drug screening and therapeutic evaluation.

## 2. Materials and Methods

### 2.1. Cell Culture and 3D Model Generation

Caucasian male Homo sapien (human) hepatocellular carcinoma cell lines (HepG2; ATCC, HB-8065, Manassas, VA, USA) were used in this study. The use of HepG2 cells is due to their low cost and ease of use, which make them ideal cells for high-throughput environments [10,41]. HepG2 cells were maintained in 75 cm^2^ culture flasks containing high-glucose DMEM medium (D6429; Sigma, St. Louis, MO, USA) supplemented with 2.5 mM L-glutamine (G7513, Merck LifeScience S.r.l., Milan, Italy), 10% Fetal Bovine Serum (F7524, FBS, Merck Life Science S.r.l., Milan, Italy), 1% penicillin/streptomycin/amphotericin (A5955, Merck Life Science S.r.l., Milan, Italy), and incubated in a humidified atmosphere containing 5% CO_2_ at 37 °C. The culture medium was replaced with fresh medium every 3 days, and the cells were subcultured. For spheroid formation and establishment of the 3D HCC model, HepG2 cells were trypsinized, counted using a hemocytometer with Trypan Blue (15,250,061, Thermo Fisher Scientific, Waltham, MA, USA), and seeded as single-cell suspensions in 96-well ultra-low-adhesion round-bottom plates (174,927, Thermo Scientific, Waltham, MA, USA) at defined densities of 2500, 5000, 10,000, and 15,000 cells/well in a final volume of 100 µL/well. Cells were allowed to spontaneously aggregate at the bottom of the wells, and spheroid formation was monitored at predetermined time points (1 day, 1, 2, and 4 weeks) using inverted bright-field microscopy (10 × objective), allowing assessment of size, compactness, and homogeneity over time.

### 2.2. Cell Viability by MTT Assay

Cell metabolic activity was assessed by the 3-[4,5-dimethylthiazol-2-yl]-2,5-diphenyltetrazolium bromide (MTT) reduction assay and used as an indirect indicator of cell viability and functional status. HepG2 cells were seeded into ultra-low attachment 96-well plates at different initial densities. Parallel 2D cultures were plated at the same cell numbers in standard tissue culture-treated 96-well plates and used as controls. To ensure a reliable comparison, 2D controls were maintained under standard culture conditions with routine passaging, and freshly plated cells were used as time-matched controls at 1, 2, and 4 weeks. At 1, 2, and 4 weeks of incubation, the medium was removed, and the spheroids were gently washed with PBS (without calcium and magnesium). Subsequently, 200 µL of MTT solution (1 mg/mL) (M6494, Thermo Fisher Scientific, Waltham, MA, USA) was added to each well and incubated for 2 h at 37 °C in 5% CO_2_. After incubation, the resulting formazan crystals were solubilized in 200 µL of DMSO, and absorbance was measured at 540 nm using a Synergy HT plate reader (BioTek Instruments, Winooski, VT, USA). All measurements were performed in triplicate. Cell viability in the 3D HCC model was expressed as a percentage relative to the absorbance values of the corresponding 2D controls seeded with the same initial cell number.

### 2.3. Liv e/Dead Staining by Confocal Microscopy

Cell viability within the 3D model was assessed using a Live/Dead Viability Kit (Thermo Fisher Scientific, Waltham, MA, USA; Cat. No. L3224), based on SYTO 9 and propidium iodide (PI) fluorescent probes. The staining solution was freshly prepared in phosphate-buffered saline (PBS) (PBS; pH 7.4; Thermo Fisher Scientific, Waltham, MA, USA; Cat. No. 10010023) to final concentrations of 5 μM of SYTO 9 and 30 μM of PI. The solution was directly added to each well containing spheroids and incubated for 30 min at 37 °C in the dark to ensure optimal staining. The Confocal Laser-Scanning Microscopy (CLSM) analysis was performed using a Leica DMIRE2 microscope equipped with a TCS SP2 laser-scanning system. SYTO 9 fluorescence (viable cells) was excited at 488 nm and detected between 500 and 550 nm; PI fluorescence (non-viable cells) was excited at 561 nm and detected between 570 and 620 nm. Images were acquired using a 20× and 63× oil immersion objective and processed with ImageJ (National Institutes of Health, Bethesda, MD, USA; https://imagej.net/ij/ accessed on 22 May 2024).

### 2.4. Gene Expression Analysis by qRTPCR

To investigate gene expression levels during the progression of the 3D HCC model, the *PCNA*, *KI67*, *AFP*, *HIF-1α*, *BBC3*, *SLC2A1*, and *LDHA* genes were analyzed using quantitative real-time PCR (qRTPCR). Total RNA was extracted using TRIzol™ Reagent (Invitrogen, Carlsbad, CA, USA; Cat. No. 15596018) according to the manufacturer’s instructions. RNA samples were quantified using an ND-1000 UV spectrophotometer (Thermo Fisher Scientific, Waltham, MA, USA). For quantitative real-time PCR, cDNA was synthesized from 1 µg of total RNA using the ImProm-II™ Reverse Transcription System (Promega, Madison, WI, USA; Cat. No. A3800). qRT-PCR was carried out in a 20 μL reaction mixture containing 1 μL of cDNA preparation, 0.5 mM of each forward and reverse primers, and 10 μL of SsoAdvanced universal SYBR1 Green supermix (2×) (2×, Bio-Rad, Hercules, CA, USA; Cat. No. 1725120) [42,43]. For this study, gene expression data were analyzed by the 2^−ΔΔ^*^Ct^* method and presented as fold change relative to day 1. Glyceraldehyde 3-phosphate dehydrogenase (GAPDH) was used as a control for normalization. Gene targets used are reported in Table 1 [34,44,45,46].

### 2.5. Immunofluorescence Analysis

Immunofluorescence staining was performed to evaluate extracellular matrix (ECM) organization in the 3D HCC model cultured for 1 and 4 weeks. Spheroids were fixed in 4% paraformaldehyde, permeabilized with 0.1% Triton X-100, and blocked in 1% bovine serum albumin (BSA). Primary antibodies targeting SPARC (osteonectin) (rabbit polyclonal, Invitrogen, Carlsbad, CA, USA; Cat. No. PA5-27287) were used. After washing with PBS, spheroids were incubated with Alexa Fluor^®^ 555-conjugated anti-rabbit IgG secondary antibody (goat polyclonal, Invitrogen, Carlsbad, CA, USA; Cat. No. A-21428) for 1 h at room temperature in the dark (1:500). Samples were then mounted onto glass microscope slides, and measurements were performed by a Leica DMIRE2 inverted microscope equipped with a TCS SP2 laser-scanning confocal system. The fluorescence excitation was set at 514 nm, while the emission was collected at 560 nm, in order to minimize the spectral overlap and laser reflection. Image acquisition was collected by using a 63× oil immersion objective. Image analysis was performed using ImageJ (National Institutes of Health, Bethesda, MD, USA; https://imagej.net/ij/ accessed on 22 May 2024).

### 2.6. Flow Cytometric Analysis

In order to evaluate cell viability, the 3D HCC model was collected at baseline (T0), and following 2 and 4 weeks of culture (T2 and T4), it was stained with a Live/Dead stain kit (Cat. Num. L23105, Invitrogen, Waltham, MA, USA). Briefly, cells were collected, washed twice in PBS, and stained with viability dye for 30 min at room temperature; cells were then washed and analyzed by flow cytometry. The apoptotic cell status of tumor cells cultured in 3D was assessed using FITC Annexin V (Cat. Num. A35110, Invitrogen, Waltham, MA, USA) and 7AAD (Cat. Num. A07704, Beckman Coulter, Brea, CA, USA) staining. Cells were washed twice in PBS and then in Annexin-binding buffer. Cells were resuspended and stained with Annexin V (FITC, Cat. Num. A35110, Invitrogen, Waltham, MA, USA) and 7AAD (Cat. Num. A07704, Beckman Coulter, Brea, CA, USA) for 15 min at room temperature, washed, and analyzed. In order to measure reactive oxygen species (ROS) in the 3D HCC model, cells were washed twice in PBS (Cat. Num. ECB4053, Euroclone, Pero, Italy) and stained with CellROX^®^ Oxidative Stress Reagents (Cat. Num. C10444, Invitrogen, Waltham, MA, USA) for 30 min at 37 °C. Cells were washed three times with PBS. Data were acquired using a FACS Symphony (A3, BD Bioscience) and analyzed by FlowJo version X software (BD Bioscience). The ANOVA test was applied to analyze the data using GraphPad Prism version VIII.

### 2.7. Fourier-Transform Infrared Spectrscopy (FTIR) Analysis

The instrument used to acquire the IR transmission spectra of the cells is the Spectrum 100 spectrometer (PerkinElmer). The apparatus is equipped with both a microscope (15× optical objective collinear with the IR beam) to select the area for spectrum acquisition via an optical imaging system with a digital camera and with a configuration that allows measurements in attenuated total reflection (ATR) mode. The stable radiation source used for the experiments is a Nernst lamp (a ceramic made of zirconium oxide with additions of yttrium oxide heated up to 1500–2000 °C). The spectrometer uses a focal plane array (FPA) mercury cadmium telluride (MCT) detector with a resolution of 2 cm^−1^. Measurements were conducted at room temperature on cells cultured on 10 × 10 mm^2^ CaF_2_ slides, in an ATR configuration. Spectra were acquired from an area of about 100 × 100 μm^2^ of the sample. Several regions were explored on each slide, and multiple spectra were obtained for each position. Spectra were obtained from fixed cell samples at various weeks after seeding on the slides. Background signals were acquired from a cell-free region of the slide and collected over the spectral range from 4000 to 650 cm^−1^, using 5 scans with a spectral resolution of 2 cm^−1^ and an acquisition time of 8 s per spectrum, all at room temperature.

For FTIR analysis, the control comparisons are based on different time points during the maturation of the same HepG2-derived 3D spheroids, as well as with respect to the initial free cells in suspension that have not yet undergone assembly and represent our starting point. This approach enables monitoring of chemical and structural changes throughout spheroid formation and maturation. Furthermore, normalization procedures were applied to FTIR data to ensure an accurate and reliable comparison between samples. This involved baseline correction and normalization to total spectral area in the investigated range in order to minimize experimental variability and focus on changes related to cellular biochemical composition and ECM components.

### 2.8. Statistical Analysis

The data were derived from three independent experiments conducted in triplicate. All the data are expressed as the means ± standard deviation. Data were analyzed using one- or two-way ANOVA followed by Bonferroni’s correction unless otherwise stated. These analyses were performed using GraphPad Prism version 8.0.1 (GraphPad Software, San Diego, CA, USA). *P*-values of 0.05 or less were regarded as significant.

## 3. Results

### 3.1. Three-Dimensional HCC Model’s Morphological and Structural Evolution

**Figure 1:**
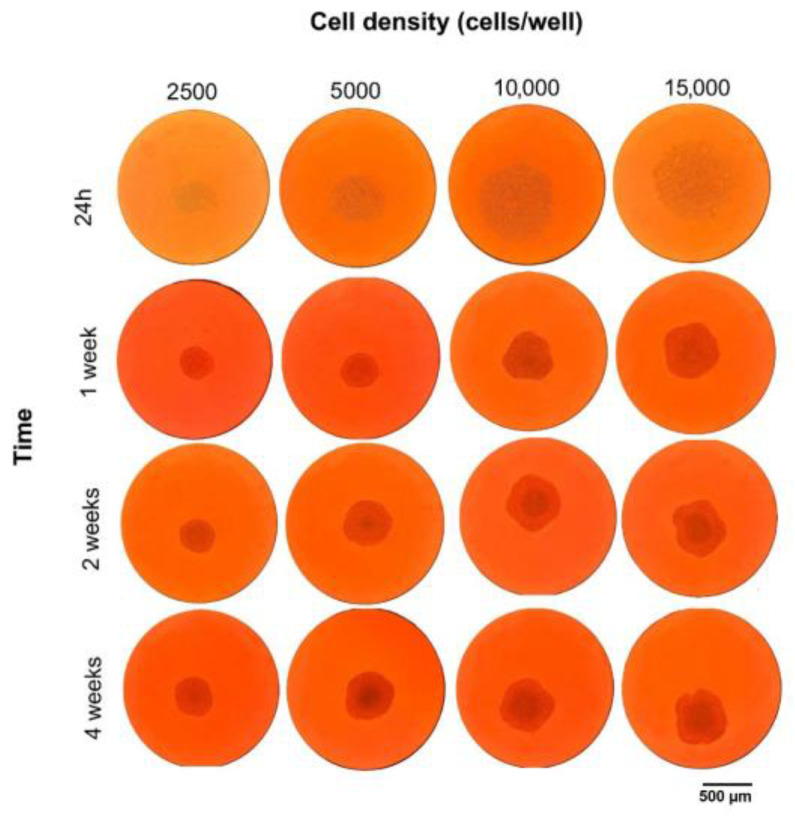
Morphological evolution of the 3D HCC model over time and at different seeding densities. Scale bar = 500 µm. The data were derived from three independent experiments conducted in triplicate. Each data point represents the mean of three replications.

HepG2 cells were seeded in 96-well cell culture plates at increasing starting densities (2500, 5000, 10,000, and 15,000 cells/well) to promote 3D HCC model formation. Growth and morphological features were evaluated at 24 h (starting point), 1, 2, and 4 weeks using bright-field microscopy at 10× magnification (Figure 1). The size of the spheroids varied with the number of cells seeded at time 0 in each well and increased with incubation time. At 24 h after seeding HepG2 cells, the onset of cell aggregation was observed in all the different initial densities. Therefore, it was taken as a starting point for the formation of three-dimensional spheroids. At week 1, spheroids with an initial density of 2500 and 5000 cells/well presented a compact and spherical morphology, in contrast to spheroids with an initial density of 10,000 and 15,000 cells/spheroid, which began to show irregularities in their structure. At the same time, all the spheroids appeared without a detectable central core, regardless of seeding density. In the second week, no structural change was observed in spheroids with an initial density of 2500 cells/well, in contrast to spheroids with an initial density of 5000 cells/well, in which size growth was observed to increase in a time-dependent manner, with the presence of an undetectable central core. In contrast, in spheroids with an initial density of 10,000 and 15,000 cells/well, the growth was found to be increasingly irregular, with a noncompact external morphology and the presence of a marked central core, suggesting the development of internal structural heterogeneity.

At week 4, marked morphological differences between the different initial densities were observed. Spheroids with an initial density of 2500 cells/well did not increase in size, remained small, and lacked a central nucleus. Spheroids with an initial density of 5000 cells/well maintained a compact and symmetrical structure, showing time-dependent growth and an increase in size. In addition, they presented a contained central core. Spheroids with an initial density of 10,000 cells/well showed external disorganization and a more prominent central nucleus. Spheroids with an initial density of 15,000 cells/well showed a highly irregular external architecture, characterized by a more pronounced central core. Based on these observations, spheroids seeded with an initial density of 5000 cells/well were selected for subsequent analysis due to their consistent morphology and structural stability over time.

### 3.2. Cell Viability by MTT Assay

**Figure 2:**
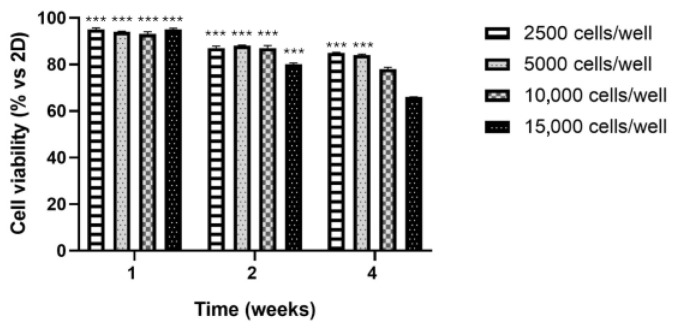
MTT assay of the 3D HCC model at 1, 2, and 4 weeks. Data are expressed as mean ± standard deviation (SD) from three independent experiments conducted in triplicate. Statistical analysis was performed using one-way ANOVA followed by Bonferroni’s correction. Significance levels are indicated as *** *p*-values <0.001.

Cell viability was evaluated using the 3-[4,5-dimethylthiazol-2-yl]-2,5-diphenyltetrazolium bromide (MTT) assay at 1, 2, and 4 weeks in HepG2 spheroids with varying initial cell densities (Figure 2). The results were compared to those obtained from traditional 2D culture systems. At week 1, all densities at which spheroids were grown showed high viability, ranging from 98% to 96% compared with 2D cultures. At week 2, all densities showed a decrease in viability, ranging from 89% (spheroids with initial densities of 2500, 5000, 10,000 cells/well) to 80% (spheroids with initial densities of 15,000 cells/well) compared with 2D cultures. At week 4, a further reduction in viability was detected in spheroids with initial densities of 2500 and 5000 cells/well (83%), but this was particularly observed in spheroids with initial densities of 10.000 cells/well (78%) and 15,000 cells/well (69%) compared with 2D cultures. In conclusion, spheroids with initial densities of 5.000 cells/well maintained viability levels of 96%, 89%, and 83% at weeks 1, 2, and 4, respectively. In line with ISO 10993-5 guidelines, viability values below 80% are generally not considered physiologically reliable for prolonged culture, further supporting the choice of the 5000 cells/well condition as the optimal model. Therefore, this condition was identified as the most stable and reproducible for performing prolonged 3D culture studies and was selected for all subsequent analyses.

### 3.3. Live/Dead Staining by Confocal Microscopy

**Figure 3:**
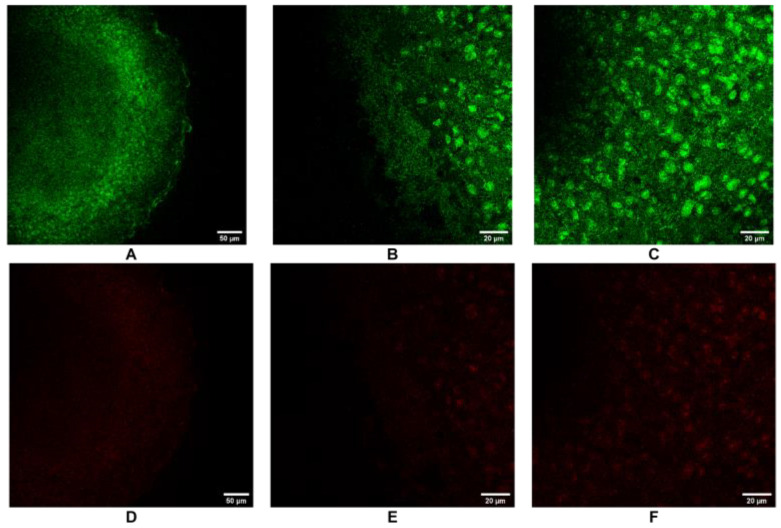
Confocal Live/Dead staining of 3D HCC model: green fluorescent live cell (**A**–**C**) and red fluorescent dead cell (**D**–**F**) images at different spheroid portions and magnification. Scale bar = 50 µm (**A**,**D**); 20 µm (**B**,**C**,**E**,**F**). The data were derived from three independent experiments conducted in triplicate. Each data point represents the mean of three replications.

CLSM images of viability assay of cells within the 3D HCC model, performed by Live/Dead staining, are reported in Figure 3. Cells were treated and analyzed at an initial density of 5,000 cells/well, which was considered the most stable and reproducible with respect to the spatial distribution of viable and non-viable cells. Green, fluorescent live cells were detected throughout the spheroid structure. A highly intensive green fluorescence, indicative of high cell viability, was observed in the peripheral region, both at 20× and 63× magnification (Figure 3A–B), and in the central region (Figure 3C) of spheroids. On the contrary, red fluorescent dead cells were poorly detectable in the peripheral region at both 20× and 63× magnification (Figure 3D–E) and in the central region nucleus (Figure 3F). The faint reddish tone observed corresponds to the basal coloration of propidium iodide rather than to a true fluorescence signal consistently, with very limited cell death detected. These results confirmed a high degree of cell preservation within the spheroids at an initial density of 5000 cells/well and at the investigated experimental times.

### 3.4. Flow Cytometric Analysis

**Figure 4:**
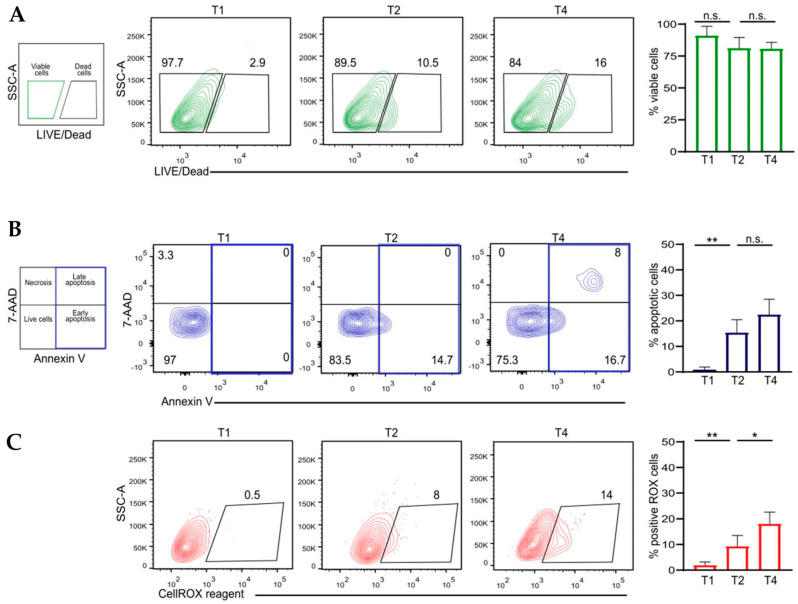
(**A**). Dot plots showing percentage of viable and dead cells in a 3D HCC model, assessed at T0, week 2 (T2), and week 4 (T4) of culture. Bars represent percentage ±SEM of viable cells (*n* = 10). (**B**). Cell phase status was analyzed by flow cytometry, measuring Annexin-FITC/7AAD labeling on the 3D HCC model at T0, T2, and T4. Bars represent the percentage of apoptotic cells (both early -Annexin V+/7AADne- and late- Annexin V+/7AAD+- apoptotic cells) (n = 10). (**C**). Analysis of ROS accumulation was assessed at T0, T2, and T4 by using CellROX reagent. Bars represent percentage ±SEM of ROS+ cells (*n* = 10). Significance levels indicated as ** *p*-values < 0.01, * *p*-values < 0.05, ns: not significant.

The analysis of viability and the cell death phase is essential for developing a reliable 3D HCC model for screening purposes. Therefore, a multiparametric flow cytometry analysis was conducted at time points T0, week 2 (T2), and week 4 (T4) to assess viability, cell death, and functionality of the 3D HCC model. The spheroids, derived from an initial seeding density, were analyzed using a Live/Dead staining assay. The results showed that the percentage of live cells, identified as Live/Dead negative cells, remained stable throughout the culture period (from T0 to T4), with an average viability exceeding 80% (Figure 4A). The cell death phase (including live, dead, and apoptotic cells) of HCC cell lines cultured in 3D models was examined using FITC-labeled Annexin V/7AAD staining. Notably, flow cytometry results revealed a gradual yet moderate increase in the early and late apoptotic populations over time, identified as Annexin V+7AAD- and Annexin V+7AAD+ cells, respectively (16%, 7%, and 8%) (Figure 4B). No necrotic cells were detected. These findings align with the observed slight decline in the live cell population. Furthermore, an analysis of ROS accumulation in the 3D tumor model revealed a slight but progressive increase in ROS+ cells over time, with values rising from 0.5% at T1 to 8% at T2 and reaching 14% at T4. Overall, the analysis of cell viability, apoptosis, and ROS accumulation in the 3D HCC model reveals a remarkably stable and consistent viability profile over time, accompanied by a gradual increase in apoptotic cell populations and oxidative stress levels. These results underscore the dynamic and physiologically relevant progression of the spheroid culture, effectively recapitulating the complexity of the evolving tumor microenvironment. Notably, maintaining the spheroid culture for up to 4 weeks does not compromise cell viability nor provoke excessive oxidative damage, establishing this model as highly suitable for long-term investigations and high-throughput screening purposes. This represents a significant advancement over existing models reported in the recent literature, offering enhanced reliability and translational relevance for cancer research and therapeutic development.

### 3.5. Gene Expression Analysis by qRTPCR

**Figure 5:**
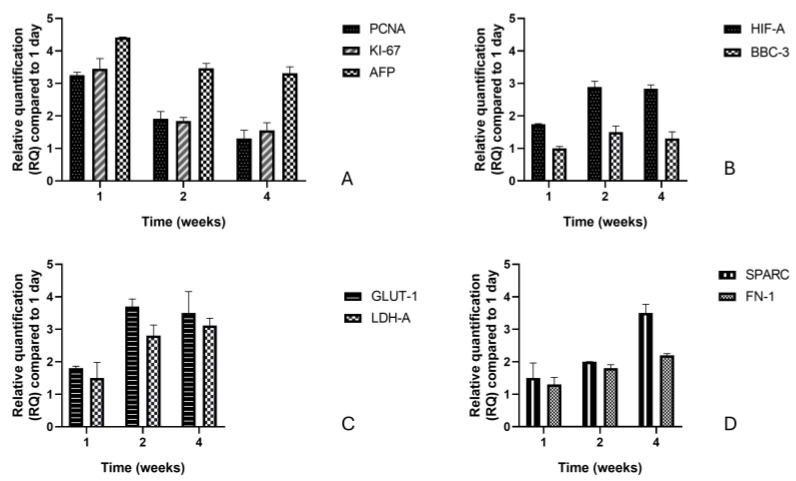
Relative expression of genes involved in the process of proliferation (**A**), apoptosis and hypoxic stress (**B**), metabolic process (**C**), and extracellular matrix (ECM) remodeling (**D**) in a period corresponding to 1, 2, and 4 weeks. The data were derived from three independent experiments conducted in triplicate. Each data point represents the mean of three replications ± standard deviation (SD).

Initially, to analyze the proliferative state and tumor characteristics of the 3D HCC model, the expression levels of *PCNA* (proliferating cell nuclear antigen), *Ki-67* (Kiel 67 antigen), and *AFP* (Alpha-Fetoprotein) were quantified [20]. Next, the pattern of genes related to hypoxic stress and apoptotic signaling was assessed by quantifying *HIF1α* (Hypoxia-Inducible Factor 1-Alpha) and *BBC3* (BCL2 Binding Component 3, also known as PUMA) [47,48,49]. In addition, the gene pattern of *SLC2A1* (GLUT1) and *LDHA* related to metabolic readjustment induced by hypoxic conditions was observed. Finally, to assess extracellular matrix (ECM) remodeling, the expression of *SPARC* (Secreted Protein Acidic and Cysteine Rich) and *FN1* (Fibronectin 1) was considered [50]. All these elements were assessed by qRT-PCR at 1, 2, and 4 weeks of culture versus day 1 (24h) (Figure 5A–D).

From the analyses, it emerges that PCNA, a protein essential for DNA replication and a marker of cell proliferation [51] showed marked upregulation at week 1 (3.25 ± 0.09-fold, compared with day 1), followed by gradual downregulation at weeks 2 (1.90 ± 0.23) and 4 (1.30 ± 0.26), compared with day 1. A similar trend was observed for Ki-67, a nuclear protein expressed in all phases of the cell cycle but absent in quiescent (G0) cells; the highest value occurred at week 1 (3.45 ± 0.32), and then it significantly decreased at week 2 (1.84 ± 0.11) and further at week 4 (1.55 ± 0.24), compared with day 1. This behavior indicates a reduction in the overall proliferation process as the spheroids matured. Additionally, AFP, a fetal plasma protein known for its pleiotropic functions and commonly used as a biomarker for hepatocellular carcinoma [52], was the most upregulated gene at week 1, showing a 4.41 ± 0.01-fold increase. Although its expression slightly declined at week 2 (3.47 ± 0.15) and week 4 (3.31 ± 0.20, relative to day 1), it remained significantly elevated. This pattern suggests that despite a reduction in proliferative activity, a poorly differentiated, tumor-like phenotype persisted over time. Overall, the results demonstrated that all three genes studied (*PCNA*, *Ki-67*, and *AFP*) decreased progressively in a time-dependent manner from day 1. HIF1α, hypoxia-inducible factor 1 [53], was slightly increased at week 1 (1.75 ± 0.01-fold), showing a clear increase at week 2 (2.89 ± 0.18), and maintaining high levels at week 4 (2.84 ± 0.12). Instead, BBC3, a pro-apoptotic member of the BCL-2 family, BH3-only, which is activated in a p53-dependent manner under stress conditions [54], remained unchanged (1.00 ± 0.06), followed by a relative increase at week 2 (1.50 ± 0.19), highlighting the activation of apoptotic pathways under prolonged oxygen deprivation conditions. At week 4, BBC3 expression slightly decreased to 1.30 ± 0.21 but remained above basal levels.

SLC2A1 (GLUT1), the primary glucose transporter that enhances glucose uptake, was upregulated in response to a key adaptation observed in tumors [55]. Since week 1, gene expression was relatively high (1.80 ± 0.10-fold), peaking at week 2 (3.70 ± 0.20) and remaining high at week 4 (3.50 ± 0.70).

Analyzing both SLC2A1 and LDHA together offers a comprehensive understanding of glycolytic pathway regulation and metabolic reprogramming in diseases. While SLC2A1 controls glucose entry into the cell, LDHA, encoding lactate dehydrogenase A, controls the downstream metabolic fate of pyruvate, together influencing energy production, biosynthesis, and cellular adaptation to hypoxia or nutrient changes. Therefore, studying both genes can provide crucial insights into metabolic changes in cancer and other conditions, potentially revealing therapeutic targets or biomarkers for disease progression and treatment response. In our study, an increase in *LDHA* was observed at week 1 (1.50 ± 0.48-fold), with a significant increase at week 2 (2.80 ± 0.33). The peak was observed at week 4 (3.12 ± 0.22).

For completeness of information, we analyze SPARC and FN-1 because both play significant roles in regulating the extracellular matrix (ECM) interactions that affect cellular metabolism and the glycolytic pathway. Specifically, SPARC encodes a cysteine-rich acidic matrix-associated protein, belonging to the extracellular matrix protein family, and contributes to ECM organization and tumor invasion [56]. The gene pattern showed a gradual increase, with values increasing from 1.50 ± 0.50-fold at week 1 to 2.00 ± 0.005 at week 2 and then peaking at week 4 (3.50 ± 0.30). The observed gene pattern shows an upregulation, indicating a continuous remodeling of the ECM during spheroid maturation. Instead, FN1 is an ECM glycoprotein involved in cell adhesion, migration, and matrix remodeling, with key roles in wound healing and metastasis formation [57]. A relative increase was observed at week 1 (1.30 ± 0.20-fold), followed by an increase at week 2 (1.80 ± 0.10), with a peak at week 4 (2.20 ± 0.10-fold). Overall, *SPARC* and *FN-1* gene expression increased in a consistent and time-dependent manner.

### 3.6. SPARC Expression and Extracellular Matrix Remodeling by Confocal Microscopy

**Figure 6:**
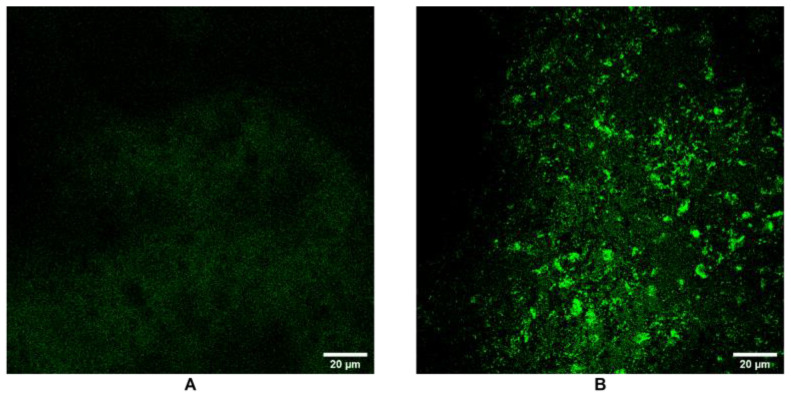
Confocal detection of SPARC in the 3D HCC model: (**A**) at 1 week and (**B**) at 4 weeks. The data were derived from three independent experiments conducted in triplicate. Each data point represents the mean of three replications.

To assess extracellular matrix (ECM) remodeling, SPARC expression was examined by immunofluorescence in the 3D HCC model cultured at 1 and 4 weeks. Confocal microscopy images showed a low-distributed green fluorescence signal at 1 week, indicating a baseline level of SPARC expression (Figure 6A). In contrast, after 4 weeks, SPARC expression was elevated, as demonstrated by the intense fluorescence predominantly localized along the spheroid structure (Figure 6B). This increase in SPARC over time suggests an active remodeling of the ECM environment during spheroid maturation.

### 3.7. FTIR Spectral Profile

**Figure 7:**
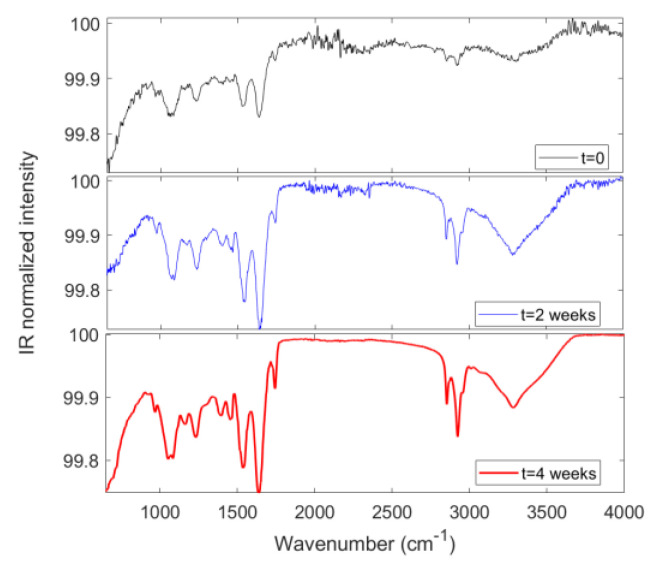
FTIR spectra of the 3D HCC model after baseline correction and normalization to total spectral area in the investigated range, in order to minimize experimental variability and focus on changes related to cellular biochemical structure.

The FTIR technique, detecting vibrational modes of chemical bonds, provides detailed insights into molecular composition and structural alterations in cells and ECM components during spheroid evolution [58,59]. We outline that the analyzed spheroids consist of HepG2 cells that self-organize into three-dimensional clusters during growth. The chosen baseline consists of the initial free cells in suspension that have not yet undergone assembly and represent our starting point. In addition, it is important to note that no exogenous scaffold or extracellular matrix (ECM) material was used to envelop the cells in the 3D model; thus, FTIR spectra, shown in Figure 7, reflect the biochemical composition of the intact 3D cellular aggregates themselves. Specifically, nucleic acids exhibit characteristic transmission bands at approximately 1080 cm^−1^ and 1230 cm^−1^, which correspond to symmetric and asymmetric phosphate (PO_2_^−^) stretching vibrations, respectively. These bands can be monitored to assess nuclear integrity and apoptosis progression, consistent with the observed increases in apoptotic markers and ROS accumulation. Moreover, protein content and secondary structure are reflected by the amide bands, primarily Amide I (around 1650 cm^−1^, mainly C=O stretching) and Amide II (around 1540 cm^−1^, primarily N-H bending coupled with C-N stretching). Changes in these spectral regions can reveal protein conformational changes or aggregation linked to cell stress mechanisms. Furthermore, lipids are characterized by C–H stretching vibrations near 2850–2950 cm^−1^ and ester carbonyl (C=O) stretching bands around 1740 cm^−1^. Shifts and/or intensity changes in these bands may indicate membrane remodeling during spheroid maturation.

The FTIR technique was also employed directly on the whole 3D spheroids to evaluate the possible formation and organization of ECM within the cellular aggregates. No external extracellular matrix scaffold was used for the 3D model growth (scaffold-free model) in order to avoid signals from both cells and scaffold materials overlapping or interfering. Moreover, no ECM extraction was performed from the 3D model prior to FTIR analysis. This choice allowed for a direct view by FTIR of cell aggregates’ intrinsic biochemistry and cell–cell interactions [60], while the traditional scaffold-based model more closely mimics the native extracellular environment but introduces additional variables due to the presence of exogenous materials [61,62]. In detail, regarding the extracellular matrix (ECM), collagen displays prominent IR bands near 1338 cm^−1^, attributed to CH_2_ wagging of proline side chains, and around 1200–1300 cm^−1^, assigned to Amide III vibrations (mainly N–H bending and C–N stretching) [59]. Glycoprotein contributions typically appear in the 1000–1150 cm^−1^ region due to C–O–C and C–O stretching vibrations. Specifically, the band splitting observed at 4 weeks in the 1000–1150 cm^−1^ region suggests that glycoproteins experience distinct local microenvironments or adopt different conformations. So, the increased expression and localized presence of ECM remodeling factors like SPARC can be correlated with changes in the intensity and position of IR bands, reflecting collagen fiber reorganization and glycoprotein [63,64]. FTIR data highlight the dynamic maturation process intrinsic to the 3D environment, which is difficult to replicate in 2D monolayers. Generally, FTIR spectra of 2D HepG2 cells usually show higher absorbance peaks in the characteristic nucleic acid regions (around 1180–1280 cm^−1^ for DNA and 1040–1140 cm^−1^ for RNA) compared to their 3D counterparts. The reduction in these nucleic acid signals likely relates to increased cell–cell interactions, the formation of multicellular spheroids, and a more physiological microenvironment that modulates nucleic acid content and structural conformation differently from flat 2D cultures [65,66]. In support, similar observations have been reported in other cancer cell types, where 3D spheroids show altered nucleic acid FTIR signals compared to 2D cultures, reflecting a closer resemblance to in vivo conditions. Simultaneously, the protein secondary structure and lipid content also change from 2D to 3D models, which indirectly influence the nucleic acid state and interactions detectable by FTIR. All of this is in good agreement with Wu et al. [67], showing that 2D vs. 3D HepG2 cultures’ functional differences include increased enzymatic activity (e.g., Pi-class Glutathione S-Transferase), hypoxia-inducible factor expression, and higher drug resistance in 3D cultures relative to 2D. These findings confirm the superior physiological relevance of 3D HepG2 models for liver research to better mimic the biological complexity of tumor cells, including nucleic acid metabolism and toxicity studies compared to conventional 2D monolayers [68]. Ultimately, changes observed in FTIR spectra correspond well with the Raman-derived biochemical markers presented and discussed in our previous paper [69] and are consistent with variations in the expression of genes involved in proliferation (PCNA, KI-67), differentiation (AFP), hypoxia (HIF-1α), and apoptosis (BBC3). This combined spectroscopic approach thus strengthens the understanding of the physiological evolution and metabolic dynamics in the 3D HCC model (cell viability, apoptosis, oxidative stress, and ECM remodeling), providing a more comprehensive molecular characterization through complementary vibrational fingerprinting, also complementing conventional assays, which provide insights into spheroid biochemical dynamics.

## 4. Discussion

This study integrates data from MTT viability assays, flow cytometry for ROS quantification, confocal microscopy for morphological and ECM remodeling assessment, and FTIR spectroscopy to monitor biochemical changes associated with hypoxia-induced oxidative stress. This multilevel analytical approach enables a comprehensive characterization of the hypoxia-driven processes affecting tumor progression in our model. The primary aim was to validate the in vitro 3D model as a physiologically relevant system for investigating ECM remodeling and oxidative stress dynamics under hypoxia. Our results consistently indicate adaptive tumor behaviors related to ROS accumulation and ECM changes over time, providing mechanistic insights aligned with the current understanding of HCC progression. Based on the current literature [37,38,39,40], the HCC developed a 3D model, demonstrating considerable strength, validity, and innovation. It was developed starting from an initial cell density of 5000 cells/well, identified as optimal for stable and viable spheroids, and it turned out to be high performing to observe the dynamic pathophysiological processes typical of HCC, such as adaptation to hypoxic stress, metabolic reprogramming, and ECM remodeling. Through the study of gene pathways, an initial transient proliferative increase followed by growth arrest was observed, associated with an elevated expression of the biomarker for hepatocellular carcinoma, confirming the maintenance of the HCC-like phenotype over time [70]. Our studies revealed an oxygen-poor environment capable of inducing ROS. This biological axis contributes to the transcriptional upregulation of glycolytic genes such as SLC2A1 (GLUT1) and LDHA, driving a metabolic shift toward aerobic glycolysis, known as the Warburg effect [71]. Importantly, the metabolic rewiring observed in our 3D model was not an isolated event but appeared to correlate with progressive ECM remodeling, as demonstrated by the upregulation of SPARC and FN1 [33,72], and confirmed by confocal microscopy and FTIR spectral changes. Flow cytometry data further supported the link between hypoxia-induced ROS production and ECM remodeling. This suggests that lactate accumulation and downstream epigenetic effects may indirectly promote ECM reorganization, fostering a more permissive microenvironment for tumor invasion. Regarding all of this, our 3D HCC model emerges as a valuable platform for dissecting the temporal and functional interplay between hypoxia, redox imbalance, glycolytic remodeling, and extracellular matrix adaptation elements (alongside the sustained elevated expression of a key HCC biomarker, confirming that the model faithfully maintains the HCC-like phenotype over time) that, when combined, form a core axis of tumor progression [26,73]. This achieved phenotypic stability is interesting, being generally critical for longitudinal studies, a further element that validates the relevance of the model in replicating tumor biology and for future testing of anti-metabolic or anti-matrix therapeutic interventions.

In summary, our scaffold-free 3D HCC model, optimized at 5000 cells/well, successfully maintains phenotypic stability for up to four weeks and recapitulates the interconnected processes of hypoxia-driven ROS accumulation, metabolic reprogramming, and ECM remodeling. This integrated axis, supported by multiparametric analyses, positions the model as a robust and translational platform for investigating HCC progression and for the future testing of targeted therapeutic interventions.

## 5. Conclusions

This study presents a robust in vitro 3D HCC model combining MTT assays, ROS quantification, confocal microscopy, and FTIR spectroscopy to comprehensively characterize hypoxia-induced oxidative stress and ECM remodeling. Starting from an optimal seeding density of 5000 cells/well, the model supports stable spheroid formation and recapitulates key pathophysiological features of HCC, including adaptation to hypoxia, metabolic reprogramming, and ECM changes. Hypoxia-induced ROS accumulation triggers upregulation of glycolytic genes (SLC2A1, LDHA), promoting a Warburg-like metabolic shift linked to progressive ECM remodeling via SPARC and FN1 upregulation. This interplay suggests lactate-driven effects, facilitating a tumor-permissive microenvironment. This developed 3D HCC model provides a powerful platform to dissect the complex interplay between hypoxia-induced oxidative stress, metabolic reprogramming, and ECM remodeling. Future studies leveraging this system could unravel novel molecular mechanisms driven by lactate accumulation, potentially identifying new therapeutic targets to disrupt the tumor-permissive microenvironment and improve HCC treatment outcomes.

## Figures and Tables

**Table 1:** Gene targets used in qRT-PCR.

Protein Name	Target Gene	Forward	Reverse
Glyceraldehyde3-phosphate dehydrogenase	***GA** **PDH***	AACAGCGACACCCACTCCTC	CATACCAGGAAATGAGCTTGACAA
Proliferating cell nuclear antigen	***PCNA***	CAAGTAATGTCGATAAAGAGGAGG	GTGTCACCGTTGAAGAGAGTGG
Proliferation marker protein KI-67	**KI-67**	GAAAGAGTGGCAACCTGCCTTC	GCACCAAGTTTTACTACATCTGCC
Alpha-fetoprotein	***AFP***	GCAGAGGAGATGTGCTGGATTG	CGTGGTCAGTTTGCAGCATTCTG
Hypoxia-inducible factor 1-alpha	***HIF1α***	TATGAGCCAGAAGAACTTTTAGGC	GATGGCAGTAGCTGCGCTGATA
Bcl-2-binding component 3	***BBC3***	ACGACCTCAACGCACAGTACGA	GCAGGAGTCCCATGATGAGATTGT
Solute Carrier Family 2, Member 1	***SLC2A1 (GLUT1)***	TTGCAGGCTTCTCCAACTGGAC	CAGAACCAGGAGCACAGTGAAG
Lactate Dehyfrogenase A	***LDHA***	GGATCTCCAACATGGCAGCCTT	AGACGGCTTTCTCCCTCTTGCT
Secreted Protein Acidic and Cysteine-Rich	***SPARC***	TGCCTGATGAGACAGAGGTGGT	CTTCGGTTTCCTCTGCACCATC
Fibronectin 1	***FN1***	ACAACACCGAGGTGACTGAGAC	GGACACAACGATGCTTCCTGAG

## Data Availability

All data supporting the findings of this study are available within the document.
